# The Effect of Household Dependency Ratio on the Mental Health of Workforce: Evidence From China

**DOI:** 10.3389/fpubh.2022.848114

**Published:** 2022-04-08

**Authors:** Dongli Fang

**Affiliations:** College of Economics, Jinan University, Guangzhou, China

**Keywords:** household dependency ratio, mental health, fertility rate, CGSS, labor force

## Abstract

Based on the Chinese General Social Survey (CGSS) 2010, this article investigates the relationship between household dependency ratio and the mental health of household workforce. The empirical results verify the negative impacts of both household old-age dependency ratio and child dependency ratio on the mental health of the workforce and find that the negative effect of old-age dependency ratio is greater than that of child dependency ratio. Moreover, the depression source of the young workforce mainly comes from the child dependency ratio, while the depression source of the older workforce comes from the old-age dependency ratio. The mental health of the workforce in poor regions is impaired by the household dependency burden, but we found no same evidence in rich regions. The old-age dependency ratio negatively affects the mental health of the workforce due to the household healthcare burden, while the child dependency ratio negatively affects the mental health of the workforce due to the education expenditure pressure. Our findings provide evidence on how household structure affects the welfare of the family, and give implications to the improvement of residents' health.

## Introduction

### Social Background of Chinese Household

**Figure 1:**
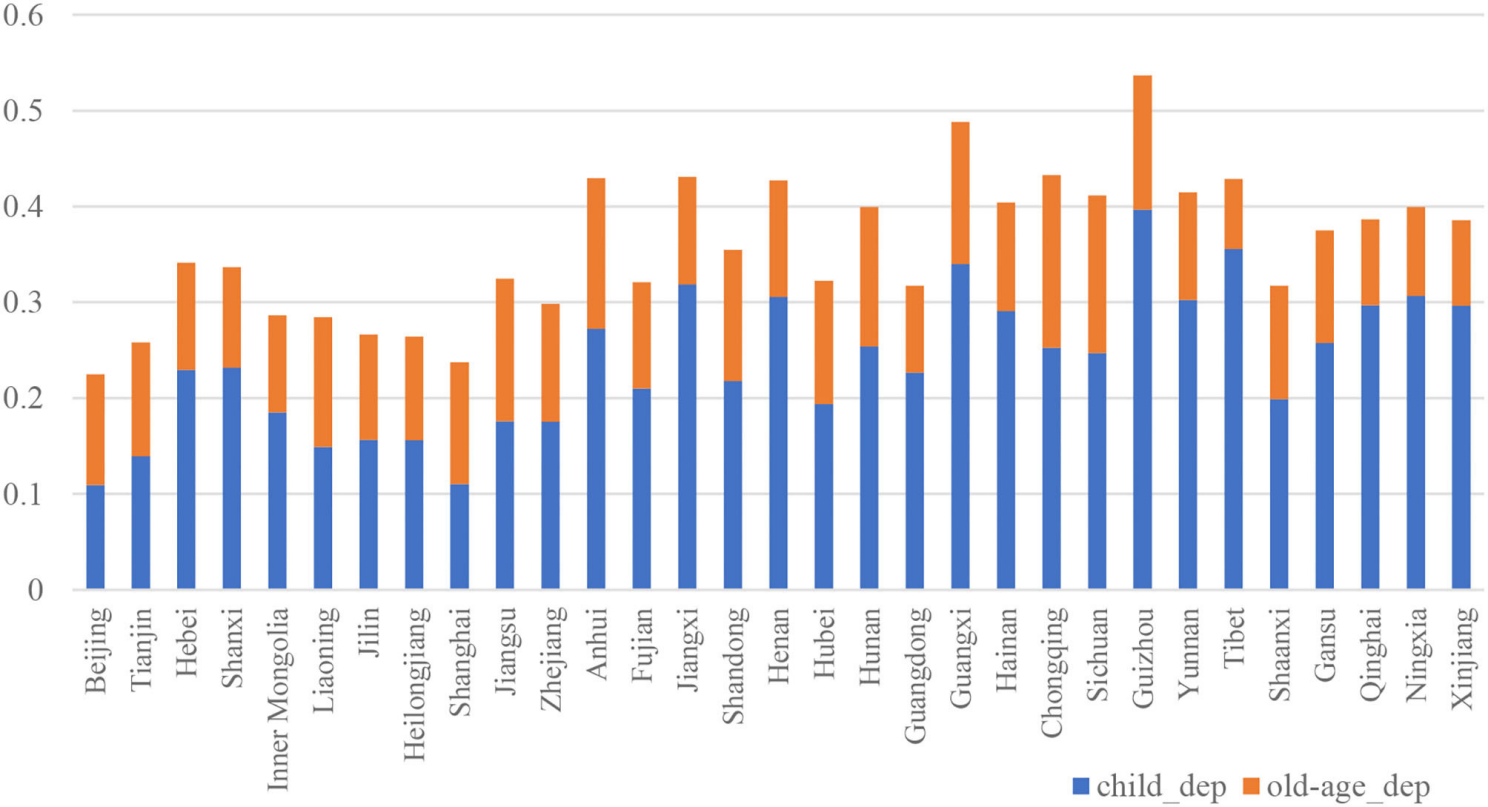
Child dependency ratio and old-age dependency ratio of each province.

According to the statistics of the National Bureau of Statistics of China, the fertility rate of China has fallen steadily since the beginning of the 1990s. At the same time, due to the huge population base and the implementation of the one-child policy, the number and proportion of the older adults population have increased rapidly, accelerating the pace of population aging. The bulletin of the Seventh National Population Census of China, which was carried out on 1 November 2020, shows that compared to the Sixth National Population Census, which was carried out 10 years ago, the share of population aged 0–14 increased by 1.35%, the share of population aged 60 and above increased by 5.44%, and the share of the population aged 15–59 decreased by 6.79%. Except for Tibet, the share of population aged 65 and above in the other 30 provinces of mainland China exceeded 7%. The Population Division of the United Nations predicts that by the year 2050, the total dependency ratio of China will be 67.3%, among which the old-age dependency ratio will be 43.6%^1^. It can be seen that the labor supply of China is decreasing and the total dependency ratio of the population is rising. Figure 1 shows the dependency ratio of each province in mainland China in 2010. Guizhou province has the highest total dependency ratio because young people emigrate to the developed province and leave their children and parents their hometown. At the same time, most of the people in Guizhou province belong to minority ethnic groups, whose fertility is not limited by the one-child policy. Beijing and Shanghai have the lowest dependency rate because they are emigration destinations for young people.

The family planning policy that was formally implemented in the 1970s which aimed to control the population changes China's household demographic structure and leads to the reduction of labor forces in the 21st century. Before the enforcement of the one-child policy, a campaign named “Later, Long, Fewer” was initiated in the 1970s, which led to the total fertility rate of China declining from 5.7 in 1969 to 2.7 in 1978 (1), and then the total fertility rate decreased steadily henceforth with the implementation of the famous one-child policy. It is estimated that the number of one-child families accounts for one-third of the total families in 2007 (2). The deficiencies of the one-child family stand out when the parents of one child enter into their old age. An adult child has to support two older adults people in a typical one-child family, which puts tremendous economic and mental pressure on that only labor force of the household, since the burdens of economic support, life caring, and spiritual consolation are imposed on the young labor force. When the adult children of the family reach childbearing age and their parents are getting old, the household dependency ratio increases further.

To solve the problem of shrinking workforce, the Chinese government gradually relaxes the control of childbirth and even encourages couples to have more children. However, these policies produce very little effect. Another plan taken by the Chinese government is to postpone retirement, but its effect needs to be observed. Muszynska and Rau (3) decompose the old-age dependency ratio into the old-age healthy dependency ratio and the old-age unhealthy dependency ratio and apply the Eurostat's population projection to anticipate the impact of population aging on the supply side of the European labor market. It is concluded that improvements in health will not compensate for the aging process on the supply side of the labor market in the long run. Nevertheless, something is better than nothing; healthy elders are able to temporally solve the problem of workforce shortage. In the long run, the best way to solve the decline of the labor force is to raise the fertility rate. However, the heavy physical and mental pressures that are put on the adult child impair the welfare of both the adult child and elderly parents and reduce the willingness of couples to have a second child.

Household is a significant component of society, and the healthy development of the household is the foundation of a healthy society. As an economic pillar of the household, the workforce plays an important role in supporting the family and faces great mental pressure. Unhealthy mental status of the workforce is not only harmful to family and society but also leads to productivity loss in the workplace (4, 5). Therefore, it is necessary to analyze the relationship between household structure and the mental health of the workforce.

### Literature Review

Scholars have investigated the effect of regional factors such as pollution (6–8), biological invasions (9), socioeconomic welfare (10), trade (11–15), macroeconomic conditions (16, 17), as well as the household factors such as social relations (18, 19), living arrangement (20–25) on residents' physical and mental health. The impact of pollution and biological on health is self-evident. As to the socioeconomic factors, Li et al. (10) find that better socioeconomic welfare is associated with a lower probability of depression symptoms among Chinese older adults, while the effect of medical facilities is the opposite. Pierce and Schott (15) find that a severe shock to the local labor market is associated with an increase in deaths of despair. They calculate the counties' exposure to the trade policy of permanent normal trade relations to China (PNTR), which was granted by the United States Congress in October 2000, and investigate its relationship to counties' mortality. It is found that the exposure to PNTR increases the death of drug overdoses. Huang et al. (26) find that the rising national income is conducive to happiness and anxiety reduction, but increases the possibility of depression disorder. Drake and Wallach (27) find that employment is beneficial to mental health. Hollingsworth et al. (16) find that macroeconomic shock has a positive impact on the overall drug death rate, which is mainly driven by rising opioid deaths. Economic pressure exerts a negative effect on the resident's mental health, which may further lead to some harmful unhealthy behaviors such as risky alcohol, smoke, and drug use (17, 28, 29).

On the household level, a number of studies investigate the impact of social relationships and living arrangements on the physical and mental health of older adults. Hughes and Waite (20) apply the Health and Retirement Study sample of Americans to study the effect of household structure on the health of people aged 51–61, finding that members of married couples living alone or with children are healthiest. The research of Weissman and Russell (24) finds that elders living with others report poor health conditions compared with those living with a spouse or partner. Ye and Chen (21) find that in China, elders living with children are in better mental health compared with those who live alone, and the medical expenditure of the older adults members of the household is higher, especially for those who live alone. The study of Gong et al. (22) shows that the physical and mental health of elders who live alone in rural China are poorer. Wang et al. (25) apply the longitudinal survey data of rural elders' welfare, which was conducted in Anhui province of China from 2001 to 2015, to investigate the effect of household structure on the health of rural elders. It is found that living with off-springs significantly decreases the mortality risk of elders in rural China, as off-springs provide elders with economic support and life caring. Chen et al. (30) find that the elders in rural areas face a higher risk of suffering from disability and illness, especially those who live alone. As to the effect of living arrangement on the other age groups, Kachi et al. (23) find that the female precarious workers who lived in single-parent households exhibit poor self-rated health. In addition, fertility is an important factor that affects people's physical and mental health. Ruppanner et al. (31) find that childbirth has a long-lasting effect on parent's time pressure and mental health. Chen and Fang (1) find that in the long run, the one-child policy has a negative effect on the mental health of elderly parents, but has no significant effect on their physical health. Since the core value of the Chinese family system is filial piety, multigeneration co-residence is common in rural China (32), which also has a health effect. Guo (33) finds that in rural China, the more the children, the higher life satisfaction of elders. Monetary support from children is the main reason for life satisfaction. Long et al. (34) find that lower fertility has a negative effect on the health condition of rural elders, but turns statistically insignificant for the health of urban elders.

Although many scholars have investigated the effect of household attributes on the physical and mental health of elders, few have studied its effect on other age groups. In fact, with economic development and improvement of social public security awareness, there are increasing concerns about the health of the aging population. However, too little attention is paid to the health of the workforce group, especially their mental health. To fill this research gap, we apply the data of the Chinese General Social Survey (CGSS) 2010 to empirically analyze the effect of the household old-age dependency ratio and child dependency ratio on the mental health of the labor force aged from 14 to 64. The first group of cohorts that are affected by the low fertility rate has come into middle age, and the fertility rate does not show an upward trend even the family planning policy liberalizes birth control. To ensure the sustainable development of society and provide implications for future population policy, it is necessary to study the effect of household structure on the mental health of the labor force.

### Contributions of the Research

Our study contributes to the following strands of literature. First, our study contributes to the understanding of the consequence of changing household structure. Some scholars have studied the effect of household structure on the savings rate (35), consumption (36, 37), and insurance purchase (38), and many studies have revealed the factors that affect the health status of children and elders (13, 22, 25, 39–41). But few scholars have been concerned about the health effect of household structure on adults of other age groups. In fact, every member of family is affected by the household structure. Household structure relates to the accumulation and allocation of family resources, which may affect the physical health and mental health of family members. We focus on the mental health of the workforce and study the effect of the household dependency ratio on it. Second, our study adds evidence to the factors that affect the residents' mental health. Living environment is the main reason to influence the residents' physical and mental health, but plenty of studies focus on the effect of ecological environment and economic environment, few studies take the household environment into consideration. The household dependency ratio affects the living condition and life quality of residents, but the research on the mental health effect of the household dependency ratio is insufficient.

## Conceptual Framework

To overcome the reduction of labor forces, the family planning policy of China is gradually being relaxed. In 2013, couples were able to have a second child if they met some requirements. In 2016, all couples were allowed to have a second child. However, the fertility rate of China did not grow. In 2020, the fertility rate further dropped to 1.3. To have a second child will increase the household dependency ratio and add burden to the household. At present, a typical couple has to take care of four elderly parents and their own children. That is, the household dependency ratio of an ordinary family affected by the one-child policy reaches 5/7, let alone the couple with more children. The higher dependency ratio, regardless of whether it was coming from children or elders, tightens the pecuniary budget of a family and decreases the life quality of family members.

On the other hand, in order to respond to the one-child policy and prepare for old-age life, the savings of households increase (35, 42). The high savings of households not only provide economic support for elderly parents but also lighten the economic burden of adult children. In addition, having fewer children frees up family resources and increases the quality of children (1), which ensures sufficient nutrition intake by children and lightens the burden of parents. Older adults have more savings to support their lives, and also have enough time and sufficient resources to improve their life quality, which may offset the negative effect of the household dependency ratio. From this perspective, downsized household is beneficial to both children and parents. Furthermore, elderly parents could help with taking care of grandchildren when adult children go out for work (32). That is, the household dependency ratio is not necessarily harmful to residents' welfare.

**Figure 2:**
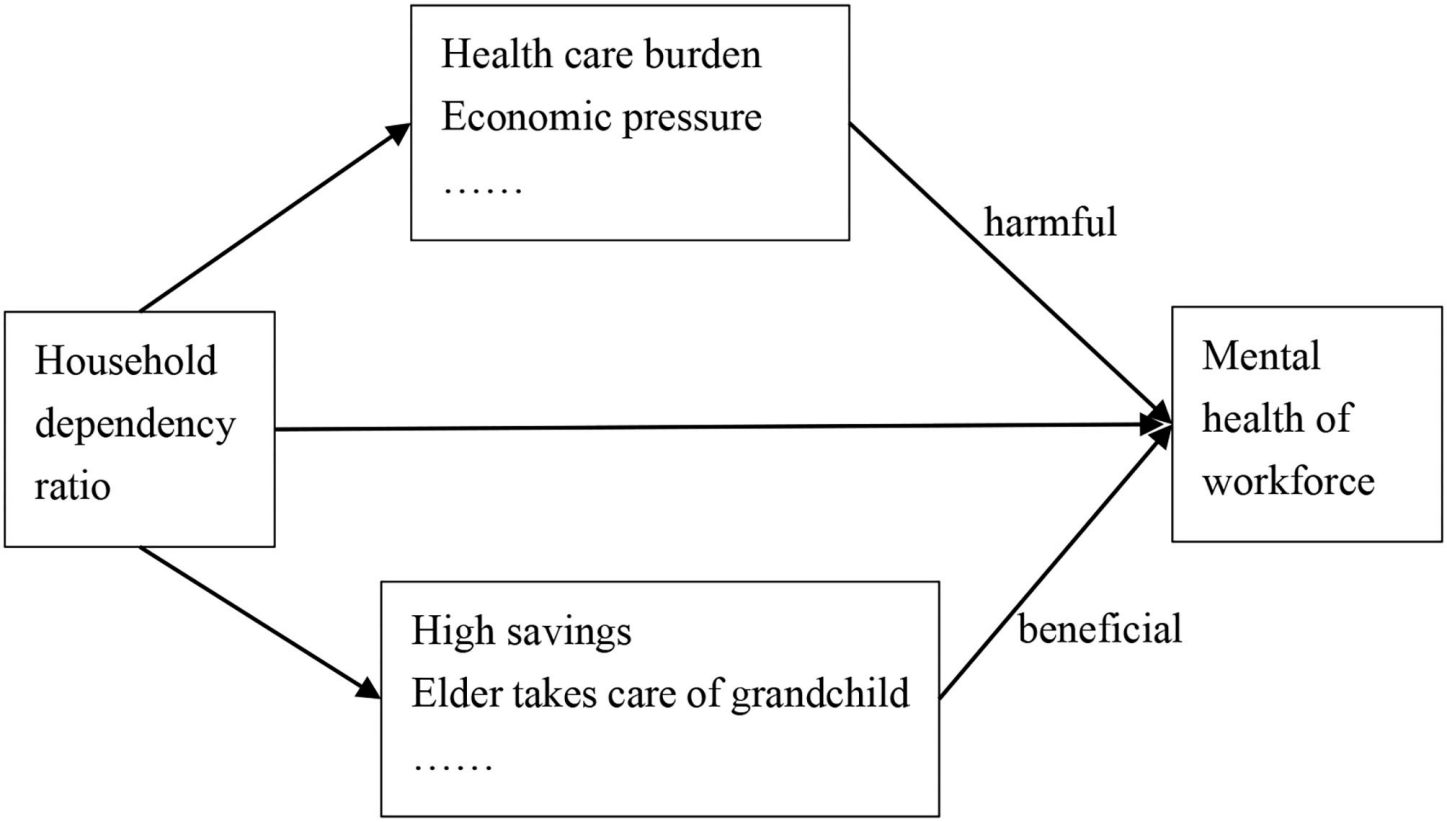
Conceptual framework of the research.

The conceptual framework of the paper is shown in Figure 2.

## Data and Empirical Strategy

The data used in this study are the CGSS in 2010, which is a national representative continuous survey project run by the Department of Sociology of the Renmin University of China. The survey in 2010 covers 480 villages/neighborhood committees in 31 provinces in mainland China including 12,000 individuals. Although the latest CGSS is the 2017 version, the 2010 CGSS provides a consumption module and a health module, the data of which are suitable for our study. The study focuses on the mental health of the workforce, so we limit the sample to those aged from 14 to 64.

### Explanatory Variable

We count the family members who are living with interviewees, categorize them into children group (aged 0–14), old-age group (aged 65 and above), and workforce group (aged 15–64). The household child dependency ratio is calculated by the number of children divided by the number of workforces, and the household old-age dependency ratio is calculated by the number of elders divided by the number of workforces. The total dependency ratio of households is the sum of the child dependency ratio and old-age dependency ratio.

### Dependent Variable

We use the mental health perception to measure the mental health of the interviewee. The question in the CSGG (2010) is “How often did you feel depressed in the past 4 weeks?” The numerical values 1–5 denote “Always,” “Frequently,” “Sometimes,” “Rarely,” and “Never,” respectively. The samples that answer “unsure/no answer” are given a null value.

### Controlled Variables

Both economic factors and non-economic factors affect the mental health of residents (43). To exclude the influence of other factors that may affect the health of people, we take the research of Li et al. (10) and Long et al. (34) for references and control individual factors such as gender, ethnic, marital status, political partisanship, age, and income, as well as household factors such as the number of family members and debt of household. Table 1 reports the summary statistics of the variables that used in this article.

**Table 1:** Summary statistics.

**Variable**	**Definition**	**Mean**	**S.D.**	**Obs.**
Depressed	Frequency of depression in the past 4 weeks			
Always	The group who answered “Always”	0.016	0.125	9,949
Frequently	The group who answered “Frequently”	0.086	0.281	9,949
Sometimes	The group who answered “Sometimes”	0.224	0.417	9,949
Rarely	The group who answered “Rarely”	0.342	0.474	9,949
Never	The group who answered “Never”	0.332	0.471	9,949
Dep_ratio	The ratio of non-workforce to the workforce	0.285	0.445	9,990
Child_ratio	The ratio of children to the workforce	0.063	0.214	9,990
Old_ratio	The ratio of elders to the workforce	0.222	0.385	9,990
Gender	Male = 1, female = 0	0.476	0.499	9,990
Ethnic	Han ethnic group = 1, other ethnic group = 0	0.903	0.296	9,990
Party	The member of Communist Party of China = 1, others = 0	0.110	0.313	9,990
Marital status	Marital status of interviewee			
Single	The group whose marital status is “single”	0.112	0.315	9,983
Cohabitation	The group whose marital status is “cohabitation”	0.002	0.040	9,983
Married	The group whose marital status is “married”	0.825	0.380	9,983
Separation without divorce	The group whose marital status is “separation without divorce”	0.004	0.067	9,983
Divorce	The group whose marital status is “divorce”	0.024	0.154	9,983
Widowed	The group whose marital status is “widowed”	0.033	0.178	9,983
Age	The age of interviewee in 2010	42.777	12.207	9,990
lnincome	The logarithm of annual income of interviewee	8.315	3.086	8,579
Home_size	The number of household members	3.031	1.372	9,990
lndebt	The logarithm of a household's debt	2.304	4.176	9,990

### Empirical Strategy

To empirically investigate the relationship between household dependency ratio and individual health, we specify the following regression model:


(1) $$\begin{array}{l} M\text{\_}health_{i}=\beta_{0}+\beta_{1}dep\text{\_}ratio_{i}+\beta_{2}Controls_{i}+\delta_{p}+\varepsilon_{i} \end{array}$$

where *M*_*health*_*i*_ is the mental health condition of the individual. The independent variable of interest is *dep*_*ratio*_*i*_, denotes the total dependency ratio, child dependency ratio, or the old-age dependency ratio of the household. *Controls*_*i*_ are a list of control variables that the literature has identified as factors that affect an individual's mental health. The δ_*p*_ is the provincial fixed effect, and ε_*i*_is the random error term.

## Empirical Results

### Baseline Results

Considering that the depression variable is ordinal, an ordered probit model is used in the main regressions. Table 2 reports the baseline regression results. When only household total dependency ratio is included, there is a significantly negative relationship between household dependency ratio and mental health of the workforce. In column (2), we add the control variables to the regressions. The coefficient of household total dependency ratio is significantly negative, indicating that household total dependency ratio is harmful to the mental health of the workforce. The workforce of the family plays an important role in supporting the family and caring for the home members, who may face tremendous stress without sufficient support from society.

**Table 2:** Baseline regression results.

	**(1)**	**(2)**	**(3)**	**(4)**	**(5)**	**(6)**
dep_ratio	−0.080***	−0.084***				
	(0.024)	(0.029)				
old_ratio			−0.158***		−0.156***	−0.138**
			(0.051)		(0.051)	(0.057)
child_ratio				−0.058**	−0.056**	−0.064*
				(0.028)	(0.028)	(0.035)
gender		0.108***				0.108***
		(0.025)				(0.025)
Ethnic		−0.070				−0.069
		(0.047)				(0.047)
Party		0.154***				0.154***
		(0.037)				(0.037)
Marital status (reference group: single)						
Cohabitation		0.344				0.337
		(0.237)				(0.236)
Married		0.113**				0.102**
		(0.049)				(0.050)
Separation		−0.302				−0.306*
		(0.184)				(0.184)
Divorce		−0.204**				−0.211**
		(0.084)				(0.085)
Widowed		−0.201**				−0.214**
		(0.083)				(0.084)
Age		−0.011***				−0.010***
		(0.001)				(0.001)
lnincome		0.020***				0.020***
		(0.004)				(0.004)
home_size		0.031***				0.031***
		(0.010)				(0.010)
lndebt		−0.035***				−0.035***
		(0.003)				(0.003)
Province FE	No	Yes	No	No	No	Yes
Obs.	9,949	8,546	9,949	9,949	9,949	8,546

Robust standard errors are reported in parentheses. ***, **, and * denote significance at 1, 5, and 10%, respectively.

To further investigate the source of negative effects, we decompose the total dependency ratio into the old-age dependency ratio and the child dependency ratio. The regression results are shown in column (3) to column (6). The core explanatory variables are added one by one. The coefficient of old-age dependency ratio is significantly negative, and its absolute value is greater than that of the child dependency ratio, indicating that the source of mental stress of the workforce mainly comes from the old-age dependency ratio. The coefficient of child dependency ratio is significantly negative at 10% statistical level, implying that household child dependency ratio is another source of individual depression. Those who were affected by the fertility policy in the 1970s would enter their forties in 2010, and become the main labor force of family and society. They have fewer siblings and face a higher household old-age dependency ratio. When aging society is increasing, elders are likely to need more governmental and other forms of social support, but the absence of this support transfers the pressure to their off-springs. The adult child should provide their elderly parents with companionship as well as financial support, implying that people have to sacrifice leisure time for elder care, which increases the mental stress of the adult child. In contrast, the household child dependency ratio is relatively stable due to the one-child policy. In addition, the 9-year compulsory education provided by the Chinese government mitigates the stress of parents. Therefore, the negative effect of child dependency ratio on the workforce is smaller.

With regard to the control variables, the results in Table 2 reveal that the mental health of men is better than women, and the mental health of the member of the Communist Party of China is better. As to the marital status of interviewee, the mental health of the married one is better than that of the single one, and the mental health of the divorce one is worse. Besides, the age of interviewee is negatively correlated with mental health, while the annual income is positively correlated with mental health.

For the household level control variables, the larger the size of the household, the better the mental health. A plausible explanation is that people have more channels to vent feelings and distract from stress when there are more family members. In addition, the more the debt of the household, the worse the mental health of the interviewee.

### Robustness Checks

We estimated the baseline regression by using the ordered probit model, in order to check the robustness of the model; we employ the OLS model to estimate. Column (1) and column (2) of Table 3 report the OLS regression results. The coefficients of core explanatory variables are hardly changed, implying that our regression results are robust even using different estimation models.

**Table 3:** Regression results for robustness checks.

	**(1)**	**(2)**	**(3)**	**(4)**
	**OLS**	**OLS**	**Happiness**	**Happiness**
dep_ratio	−0.080***		−0.090***	
	(0.028)		(0.031)	
old_ratio		−0.133**		−0.142**
		(0.054)		(0.059)
child_ratio		−0.061*		−0.071*
		(0.032)		(0.037)
Control variables	Yes	Yes	Yes	Yes
Province FE	Yes	Yes	Yes	Yes
Obs.	8,546	8,546	8,572	8,572

Robust standard errors are reported in parentheses. ***, **, and * denote significance at 1, 5, and 10%, respectively.

In the baseline regression, we employed the depression perception of the interviewee as our dependent variable. To ensure the robustness of our conclusions, we replace the dependent variable with self-rated happiness. The question in the CSGG (2010) is “Generally speaking, do you think you are happy?”. The numerical values 1–5 denote “Always,” “Frequently,” “Sometimes,” “Rarely,” and “Never,” respectively. The samples that answer “unsure/no answer” are given null values. The regression results are shown in columns (3) and (4) of Table 3. After changing the dependent variable, the coefficients of core explanatory variables change a little, which indicates that our conclusions are robust.

There may be endogeneity problems because of reverse causality, measurement bias, and omission of relevant variables (43). The household dependency ratio, especially the old-age dependency ratio, is not decided by people. Furthermore, the Chinese family planning policy was not relaxed in 2010, indicating that the child dependency ratio is not completely determined by the household. As a result, the household dependency ratio is a pre-determined variable to the mental health of individuals, which could not lead to reverse causality. With regard to the measurement bias, we take the prevailing method to calculate the household dependency ratio (38) and use two measurements to assess the mental health of the workforce. For the endogenous problems caused by the omission of relevant variables, we control the individual-level and household-level variables that may affect mental health, and also control the provincial fix effect.

### Heterogeneous Analysis

The sources of stress faced by different groups of people might be heterogeneous, and the dependency burden of the household may transfer to society with the improvement of public resources and social services. Consequently, the mental health effect of the household dependency ratio may be heterogeneous for different age groups or regions.

#### Age Heterogeneous Effect

In China, 35 years is the threshold for many careers. Therefore, we take 35 years as a boundary and divide the sample into two age groups. Table 4 reports the regression results. The coefficients of household dependency ratio of both age groups are significantly negative, indicating that the household dependency ratio impairs the mental health of the workforce. To study the source of depression, we further estimate the effect of child dependency ratio and old-age dependency ratio. As shown in columns (3) and (4) of Table 4, the source of depression for the young group mainly comes from the child dependency burden, while the source of depression for the older group mainly comes from the old-age dependency burden. For the group of workforces that aged below 35, their parents are young and they do not need to spend much time to take care of them. However, they have to spend time taking care of their children, which reduce their leisure time and lead to depression. In contrast, the older group faces the pressure from the old-age dependency. When people get older and their children reach school age, compulsory education provided by the Chinese government alleviates the child dependency stress of them, whereas their old-age dependency stresses increase. In China, the family-supporting model is the basic model for older adults care. There are few services or products for older adults care in the market, the nursing homes are insufficient compared to the large quantity of older adults, and the quality of them needs to be improved (44), so the family is the main provider of life caring and financial support for elders. Thus, the depression of older workforces mainly comes from the old-age dependency ratio.

**Table 4:** Regression results for age heterogeneous effect.

	**(1)**	**(2)**	**(3)**	**(4)**
	**Below 35**	**35 and above**	**Below 35**	**35 and above**
dep_ratio	−0.153**	−0.068*		
	(0.062)	(0.035)		
old_ratio			−0.136	−0.135**
			(0.131)	(0.064)
child_ratio			−0.157**	−0.038
			(0.070)	(0.042)
Control variables	Yes	Yes	Yes	Yes
Province FE	Yes	Yes	Yes	Yes
Obs.	2,098	6,448	2,098	6,448

Robust standard errors are reported in parentheses. **, and * denote significance at 5, and 10%, respectively.

#### Regional Heterogeneous Effect

Due to the Hukou system, internal migrations are limited, as social welfare is only available for the citizens with local Hukou. However, in order to make more money, many people still migrate to the developed region without considering the social welfare and leave their children and elderly parents in their hometown. The labor forces who stay in the hometown face higher dependency pressure. In addition, the inequality of public resources and services for the non-workforces among provinces enlarge the difference of pressure that putting on the household workforce. Therefore, we take the median value of GDP per capita of the 31 provinces as the boundary and categorize the provinces into rich and poor groups. Table 5 presents the regression results. The negative relationship between household dependency ratio and mental health of the workforce is insignificant in rich provinces, but significant in poor provinces, suggesting that the development of economic may ease the pressure of household dependency on the workforce.

**Table 5:** Regression results for regional heterogeneous effect.

	**(1)**	**(2)**	**(3)**	**(4)**
	**Rich**	**Poor**	**Rich**	**Poor**
	**provinces**	**provinces**	**provinces**	**provinces**
dep_ratio	−0.032	−0.122***		
	(0.045)	(0.039)		
old_ratio			−0.031	−0.291***
			(0.075)	(0.085)
child_ratio			−0.033	−0.079*
			(0.057)	(0.044)
Control variables	Yes	Yes	Yes	Yes
Province FE	Yes	Yes	Yes	Yes
Obs.	4,732	3,424	4,732	3,424

Robust standard errors are reported in parentheses. ***, and * denote significance at 1 and 10%, respectively.

### Mechanism Analysis

So far, we have verified the negative effect of the household dependency ratio on the mental health of the workforce, but haven't found the channel through which it takes effect. Next, we will investigate the influence mechanism of the household dependency ratio. To identify the specific influence mechanism of household dependency ratio, we take the method of Hayes (45) as a reference and construct a mediation model. The mediation model is specified as follows:


(2) $$\begin{array}{l} {Med}_{i}=\alpha +\lambda_{1}dep\text{\_}{ratio}_{i}+\lambda_{2}Controls+\varepsilon_{i} \end{array}$$

(3) $$\begin{array}{l} M\text{\_}{health}_{i}=\alpha +\lambda_{1}dep\text{\_}{ratio}_{i}+\lambda_{2}{Med}_{i}+\lambda_{2}Controls+\varepsilon_{i} \end{array}$$

According to the mediation model, the coefficient of a core explanatory variable in equation (1) is the total effect of the household dependency ratio. *Med* in equation (2) is the mediating variable, its coefficient is the effect of household dependency ratio on mediating variable. The coefficient γ_2_ in equation (3) is the effect of mediating variable on the mental health of the workforce, and the coefficient is the direct effect of household dependency ratio on the mental health, and the product of and γ_2_is the mediating effect.

Since the influence mechanism of the child dependency ratio and old-age dependency ratio is different, we separately analyze their influence channels. The mediating variables that are used in this paper are the healthcare burden of the household and the stress of education expenditure. The question about the care burden in the survey is “Do you have any family members who need care because of long-term physical and mental illness, disability, or old age and infirmity?” The answer “yes” is given 1 and “no” is given 0. Column (1) and column (2) in Table 6 present the regression results with care burden as mediating variable. It is shown that the household old-age dependency ratio is positively correlated with care burden. When the household care burden is added in the regression model, the coefficient of old-age dependency ratio turns insignificant suggesting that the household care burden fully mediates the relationship between old-age dependency ratio and the mental health of the household workforce. That is, the household old-age dependency ratio affects the mental health of the workforce by the channel of household care burden. This mechanism also explains why the household old-age dependency ratio significantly affects the mental health of the older workforce but has an insignificant impact on the young workforce.

**Table 6:** Mechanism analysis.

	**(1)**	**(2)**	**(3)**	**(4)**
	**Care**	**M_health**	**Edu_exp**	**M_health**
old_ratio	0.867***	−0.099	−0.121**	−0.143**
	(0.126)	(0.094)	(0.060)	(0.057)
child_ratio	0.028	−0.018	0.104***	−0.061*
	(0.076)	(0.062)	(0.032)	(0.035)
Med		−0.221***		−0.051***
		(0.045)		(0.008)
Control variables	Yes	Yes	Yes	Yes
Province FE	Yes	Yes	Yes	Yes
Obs.	2,843	2,832	8,474	8,446

Robust standard errors are reported in parentheses. ***, **, and * denote significance at 1, 5, and 10%, respectively.

The CGSS 2010 provides information about the various household expenditures and the corresponding pressures. One of the most important expenses for raising children is education, so we use the education expenditure pressure as the mediating variable to investigate the influence channel of the child dependency ratio. In the CGSS survey, the interviewee is asked “How much pressure does education expenditure put on your family?” The answer ranges from 1 to 5. The larger the value, the greater the pressure. As shown in column (3) of Table 6, the household child dependency ratio is positively correlated with the education expenditure pressure, while the old-age dependency ratio is negatively correlated with it. Although the compulsory education policy reduces the burden of household child dependency, adults still have to look after the life of children and educate them. However, the elders could share some of the education burden. As mentioned above, with the implementation of the family planning policy, elderly parents save more money to prepare for their retirement life. The savings of elderly parents provide economic support for themselves and their adult children lighten the economic burden of the household. Moreover, elderly parents can help look after and educate young children, which ease the stress of the workforce. When education expenditure pressure is added in the regression, as shown in column (4) of Table 6, its coefficient is significantly negative, and the coefficients of both child dependency ratio and old-age dependency ratio are significantly negative, implying that the effects of both dependency ratio are partially mediated by the education expenditure pressure. The indirect effect of the child dependency ratio on the mental health of the workforce is 0.005, accounting for 7.5% of the total effect. In contrast, the old-age dependency ratio reduces the mental stress of the workforce by alleviating the education expenditure pressure.

## Conclusion

The family planning policy that was implemented in the 1970s has lowered the fertility rate of society, leading to the reduction of the household workforce. Nowadays, the household workforce faces the heavy burden of supporting elderly parents and raising underage children. With the continuous decline of the fertility rate, the Chinese government has relaxed the family planning policy and encourages couples to have a second child. But the fertility rate did not rise. The heavy household dependency burden takes up too many family resources, the household workforce has no more spare energy to take care of themselves, which may result in depression. Therefore, to deal with the continuous shrinking of the workforce and to ensure sustainable economic development, it is necessary to study the effect of the household dependency ratio on the workforce.

It is found that the household dependency ratio is negatively correlated with the mental health of the workforce, and the negative effect of the old-age dependency ratio is greater than that of the child dependency ratio. The source of mental stress faced by the young workforce mainly comes from the child dependency ratio, while the source of mental stress of the older workforce mainly comes from the old-age dependency ratio. The negative effect of the household dependency ratio on mental health is insignificant in rich regions but is significant in poor regions. The mechanism analysis shows that old-age dependency ratio negatively affects the mental health of the workforce by the household care burden, but positively affects the mental health of the workforce by reducing the education expenditure pressure. However, the final effect of the old-age dependency ratio is negative. Except for directly influencing the mental health of the workforce, the child dependency ratio also indirectly affects it by increasing the education expenditure pressure.

Although we tried our best to improve the causal recognition of the research, there are limitations due to the availability of data. On one hand, some adults do not live with their elderly parents, but they live nearby and take care of each other. This type of household does not record in the CGSS, which may lead to the underestimation of the household dependency ratio. On the other hand, due to the emigration of the young labor force, especially in the developing regions, the household dependency ratio of some regions is very high. The workforce who stays with the children and the elders is facing heavy life caring burden while his/her economic burden is lightened because of the economic support from the one who goes out for work. We cannot estimate the mental impact of this economic support and may overestimate the mental health effect of the household dependency ratio. Even though there are limitations, the conclusion of the research is still valid. Because the family members who live with the young and the elders undertake most of the life caring and spiritual consolation work, they are most affected by the household dependency ratio.

The conclusion of the research has the following implications. First, the structure of the current Chinese household puts much care pressure on the working-age adults of the household, and impairs their mental health. To ensure the sustainable economic development and cope with the problems that accompany the shrinking workforce, it is necessary to pay more attention to the welfare of the labor force and transfer some of the household dependency ratios to society. In China, family support is the basic model for older adults care, and the old-age dependency ratio is the main source of depression in the workforce. Worsened mental health of the household workforce is harmful to every member of the family. In the future, the family-supporting model should be combined with the nursing home, and more public resources should be invested in the social older adults support project. Second, to release the mental pressure of the household workforce, schools should provide more free or low price after-class services, especially in the developing regions. The education expenditure is one of the sources that impairs the mental health of the household workforce. If schools could share a part of the education burden, the mental stress of the household workforce will be lightened. Third, more public service for the non-working population is needed. According to the empirical result, healthcare for family members is the depression source of the workforce. To solve this problem, more for-profit and non-profit healthcare services are needed, and the social insurance system needs to be further improved.

## Data Availability Statement

The datasets presented in this study can be found in online repositories. The names of the repository/repositories and accession number(s) can be found below: http://cnsda.ruc.edu.cn/.

## Author Contributions

The author confirms being the sole contributor of this work and has approved it for publication.

## Conflict of Interest

The author declares that the research was conducted in the absence of any commercial or financial relationships that could be construed as a potential conflict of interest.

## Publisher's Note

All claims expressed in this article are solely those of the authors and do not necessarily represent those of their affiliated organizations, or those of the publisher, the editors and the reviewers. Any product that may be evaluated in this article, or claim that may be made by its manufacturer, is not guaranteed or endorsed by the publisher.

## Footnotes

^1^https://population.un.org/wpp/, accessed in December, 2021.
